# Cerebrospinal fluid mitochondrial DNA in neuromyelitis optica spectrum disorder

**DOI:** 10.1186/s12974-018-1162-0

**Published:** 2018-04-27

**Authors:** Kazuya Yamashita, Makoto Kinoshita, Katsuichi Miyamoto, Akiko Namba, Mikito Shimizu, Toru Koda, Tomoyuki Sugimoto, Yuki Mori, Yoshichika Yoshioka, Yuji Nakatsuji, Atsushi Kumanogoh, Susumu Kusunoki, Hideki Mochizuki, Tatsusada Okuno

**Affiliations:** 1 Department of Neurology, Osaka University Graduate School of Medicine, D4, 2-2 Yamadaoka, Osaka, 565-0871 Japan; 2 Department of Neurology, Osaka General Medical Center, Osaka, Japan; 3 Department of Neurology, Kinki University Graduate School of Medicine, Osaka, Japan; 4 National Center of Neurology and Psychiatry, Tokyo, Japan; 5 Department of Mathematics and Computer Science, Kagoshima University Graduate School of Science and Technology, Kagoshima, Japan; 6 Center for Information and Neural Networks (CiNet), National Institute of Information and Communications Technology (NICT), Osaka University, Osaka, Japan; 7 Department of Neurology, Toyama University, Toyama, Japan; 8 Department of Respiratory Medicine, Allergy and Rheumatic Diseases, Osaka University Graduate School of Medicine, Osaka, Japan

**Keywords:** Mitochondrial DNA, NMOSD, Innate immunity

## Abstract

**Background:**

Neuromyelitis optica spectrum disorder (NMOSD) is an inflammatory disease of the central nervous system. Although complement-dependent astrocyte damage mediated by anti-aquaporin 4 autoantibody (AQP4-Ab) is well acknowledged to be the core of NMOSD pathogenesis, additional inflammatory cascades may contribute to the establishment of lesion formation. Thus, in this study, we investigated the possible pathogenic role of immune-reactive mitochondrial DNA (mtDNA) in cerebrospinal fluid (CSF) of NMOSD patients.

**Methods:**

Using quantitative polymerase chain reaction, we measured extracellular mtDNA levels in CSF of NMOSD patients positive for AQP4-Ab. Patients with multiple sclerosis or other neurological diseases were examined as controls. Pre- and post-treatment extracellular mtDNA levels were also compared in the NMOSD group. Extracellular mtDNA release from human astrocytes was analyzed in vitro utilizing NMOSD sera, and interleukin (IL)-1β production was measured in supernatants of mixed glial cells stimulated with DNA fraction of CSF derived from NMOSD patients. Furthermore, specific innate immune pathways mediating the IL-1β production by mtDNA were investigated in peripheral blood mononuclear cells with selective inhibitors of Toll-like receptor 9 (TLR9) and NOD-like receptor protein 3 (NLRP3) inflammasomes.

**Results:**

Extracellular mtDNA level was specifically elevated in acute phase of NMOSD CSF. In vitro studies provided the evidence that mtDNA is released from human astrocytes by NMOSD sera. In addition, DNA fraction isolated from NMOSD CSF promoted secretion of IL-1β from mixed glial cells. Selective inhibition of TLR9 and NLRP3 inflammasomes revealed that mtDNA-mediated IL-1β production depends on specific innate immune pathways.

**Conclusion:**

Extracellular mtDNA is specifically elevated in the CSF of patients with acute phase NMOSD, and mtDNA released by AQP4-Ab-mediated cellular damage elicits the innate immune cascades via TLR9 and NLRP3 inflammasomes pathways. Our study highlights mtDNA-mediated innate immune pathways as a novel therapeutic target for future treatment of NMOSD patients.

**Electronic supplementary material:**

The online version of this article (10.1186/s12974-018-1162-0) contains supplementary material, which is available to authorized users.

## Background

Neuromyelitis optica spectrum disorder (NMOSD) is an inflammatory disorder of the central nervous system (CNS) that predominantly affects the spinal cord and optic nerves [1]. The pathogenic role of anti-aquaporin 4 autoantibody (AQP4-Ab) has been demonstrated in previous studies [2–4]. Complement-dependent cytotoxicity and antibody-dependent cell-mediated cytotoxicity have been reported in vitro [5], and passive transfer of AQP4-Ab results in the formation of lesions pathologically similar to those observed in NMO patients [2–4].

Although the central role of AQP4-Ab in the pathogenesis of NMOSD has been firmly established, inflammatory cytokine production is essential in augmenting neuroinflammation in NMOSD. Indeed, a previous study demonstrated a synergistic effect of the inflammatory cytokine interleukin (IL)-1β in promoting NMOSD pathology and further revealed elevated production of IL-1β in affected lesions of NMOSD patients [6]. IL-1β is also present at higher levels in the acute cerebrospinal fluid (CSF) of patients with NMOSD than in those with multiple sclerosis (MS) [7]. However, the mechanism by which IL-1β production is elevated in NMOSD remains unknown.

Extracellular mitochondrial DNA (mtDNA) is released from damaged cells and promotes inflammation in various diseases [8, 9]. Aberrantly released mtDNA is a potent stimulator of innate immune system, represented by Toll-like receptor 9 (TLR9) and the inflammasome pathway, leading to further production of IL-1β [9]. In addition, recent studies demonstrated distinct activation pathways of inflammasome between mouse and human [10, 11].

Thus, in this study, we sought to determine whether mtDNA is present in CSF of patients with NMOSD, and the role of mtDNA in accelerating the innate immune cascades that establish NMOSD pathology by utilizing human-derived astrocytes and peripheral blood mononuclear cells (PBMCs).

## Methods

### Patient information and sample collection

CSF was obtained from patients with AQP4-Ab-positive NMOSD in relapse phase (*n* = 26) or post-treatment phase (*n* = 20), MS in relapse phase (*n* = 18), or other neurological diseases (ONDs, *n* = 50). All NMOSD subjects were diagnosed according to the 2015 NMOSD diagnostic criteria [1], and all MS patients fulfilled the 2010 McDonald criteria [12]. ONDs included Guillain-Barré syndrome (GBS) (*n* = 8), amyotrophic lateral sclerosis (ALS) (*n* = 15), idiopathic normal pressure hydrocephalus (iNPH) (*n* = 9), cervical spondylosis (*n* = 7), and somatic symptom disorders (*n* = 11). For the comparison of mtDNA levels among patients with similar CSF cell counts, patients with aseptic meningitis (*n* = 2), GBS (*n* = 1), and neuropsychiatric systemic lupus erythematosus (*n* = 1) were selected as a control group.

Relapse of NMOSD was defined as a sudden appearance of new neurological symptoms, and CSF during the relapse phase was collected before or within 24 h from the start of treatment (high-dose intravenous methylprednisolone or plasmapheresis). CSF in post-treatment phase was collected after symptoms resolved following treatment for the acute phase.

Sera were obtained from AQP4-Ab-positive NMOSD patients (*n* = 12) from 30 to 71 years of age (median, 43.5 years) and healthy control subjects (*n* = 7) from 25 to 60 years of age (median 39.3 years).

Informed consent was obtained from each patient. This study was approved by the ethics committee of Osaka University Hospital (permit number 12091-6).

### Quantitative polymerase chain reaction analysis

The amount of human mtDNA was measured by quantitative polymerase chain reaction (qPCR). Primers were as follows: mtDNA gene encoding cytochrome C oxidase 3 (*COX3*; forward 5′-ATGACCCACCAATCACATGC-3′, reverse 5′-ATCACATGGCTAGGCCGGA-3′), NADH dehydrogenase 1 (*ND1*; forward 5′-ATACCCATGGCCAACCTCCT-3′, reverse 5′-GGGCCTTTGCGTAGTTGTAT-3′), NADH dehydrogenase 6 (*ND6*; forward 5′-CCCCTGACCCCCATGCCTCA-3′, reverse 5′-GCGGTGTGGTCGGGTGTGTT-3′), and cytochrome B (*CYTB*; forward 5′-ATGACCCCAATACGCAAAAT-3′, reverse 5′-CGAAGTTTCATCATGCGGAG-3′) [8, 13]. Amplifications were performed on an Applied Biosystems (Carlsbad, CA, USA) 7900HT with SYBR Green (Takara Bio, Kusatsu, Japan). Standard curves were prepared by serial dilution of a plasmid containing each sequence, synthesized by Nihon Gene Research Laboratories (Sendai, Japan).

All PCR reactions were tested by melting curve analysis. The melting curves of the PCR reaction product from each primer combination were similar in all samples. Sequencing analysis was performed for each primer set to confirm amplification of the correct sequence.

### Mice

C57BL6/J mice were purchased from the Charles River Laboratories Japan (Yokohama, Japan). All of the experimental procedures were approved by the Animal Care and Use Committee of Osaka University School of Medicine.

### Cell cultures

HEK293 cells were transfected with M23-human AQP4 expression plasmids (EX-A0762-M02, GeneCopoeia, Rockville, MD, USA) using FuGENE (Roche Diagnostics, Tokyo, Japan). HEK293 cells transfected with or without human AQP4 were cultured in Dulbecco’s Modified Eagle’s Medium (DMEM) containing 10% fetal bovine serum (FBS) and 1% penicillin–streptomycin. Cells were seeded in 96-well culture plates and stimulated at 80% confluence.

Mixed glial cultures were prepared from P1 mouse pups as previously described [14]. Cells were seeded in 96-well culture plates and incubated in DMEM containing 10% FBS and 1% penicillin–streptomycin. The cells were stimulated after 14 days, when the cultures contained approximately 20% microglia.

Human astrocytes, purchased from ScienCell Research Laboratories (Carlsbad, CA, USA), were cultured on poly-L-lysine-coated T-75 flasks in supplemented astrocyte medium (ScienCell Research Laboratories). At 90% confluence, cells were passaged into 96-well culture plates and stimulated at 80% confluence.

PBMCs were cultured in RPMI 1640 containing 10% FBS, 1% penicillin–streptomycin, and 0.5% 2-mercaptoethanol. Cells were seeded at 1 × 10^6^ cells per well in 96-well plates and stimulated with the indicated compounds.

### Quantification of mtDNA

CSF was centrifuged at 2000×*g* at 4 °C for 10 min. DNA was extracted from 100 μL of supernatants using the DNA extractor SP kit (Wako, Osaka, Japan) and reconstituted in 20 μL TE buffer. Total amount of DNA was measured by the NanoDrop 2000c spectrophotometer (Thermo Fisher Scientific, Waltham, MA, USA).

HEK293 cells or human primary astrocytes were seeded in 96-well culture plates and stimulated with sera from NMOSD patients or healthy control subjects at 1:5 dilution at 37 °C for 10 h. Culture supernatants were collected and centrifuged at 2000×*g* at 4 °C for 10 min. DNA was extracted from the supernatants using the DNA extractor SP kit. Isolated DNA was used for measurement of mtDNA by qPCR as described above.

To confirm the specificity of the amplified DNA, several different pairs of primers for mitochondrial genes, encoding *COX3*, *ND1*, *ND6*, and *CYTB* were used as previously described [8, 13].

### IL-1β assay

Mixed glial cells were stimulated with DNA fractions containing abundant mtDNA. Specifically, we used DNA fractions from 100 μL CSF from NMOSD or OND patients, or the DNA fraction released from AQP4-expressing HEK293 cells stimulated with sera from NMOSD patients or healthy control subjects. After 48 h of incubation, supernatants were collected and centrifuged at 2000×*g* at 4 °C for 10 min.

PBMCs were pretreated with a TLR9 inhibitor, ODN2088 (4 μM, Miltenyi Biotec, Bergisch-Gladbach, Germany) and a NOD-like receptor protein 3 (NLRP3) inhibitor, and MCC950 (0.1 μM, Cayman Chemical, Ann Arbor, MI, USA) for 1 h and then treated with the DNA fraction from AQP4-expressing HEK293 cells stimulated with sera from NMOSD patients. After 10 h of incubation, supernatants were collected. IL-1β in supernatants was measured using the Cytometric Bead Array (BD Biosciences, San Jose, CA, USA).

### Statistics

**Fig. 1:**
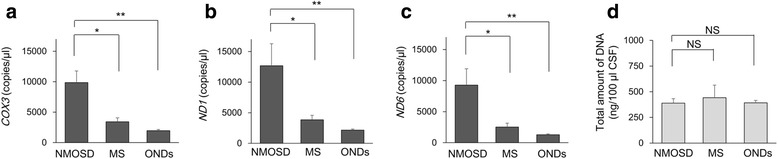
Specific elevation of CSF mtDNA in patients with NMOSD. mtDNA levels in CSF were significantly higher in patients with NMOSD in acute phase than in patients with MS in acute phase and other neurological diseases (GBS, ALS, iNPH, cervical spondylosis, and somatic symptom disorders) (**a**–**c**). The total amount of DNA was not significantly different among these groups (**d**). The data are expressed as means ± SEM. *p < 0.05; **p < 0.01; NS not significant (*P* ≥ 0.05)

Statistical analyses were performed by Mann–Whitney *U* test or Kruskal–Wallis test to compare two or more independent groups and by Wilcoxon signed-rank test to compare two paired groups in SPSS software version 14 (SPSS, Chicago, IL, USA). Holm–Bonferroni-corrected *p* values were used for pairwise comparisons after Kruskal–Wallis test (Figs. 1a–c, 3b, and 4b). We analyzed influence of imbalance on age and sex using analysis of covariance (ANCOVA) (Fig. 1). It did not reveal significant effects of CSF mtDNA levels. A value of *p* < 0.05 was considered significant. The data are presented as means ± SEM.

## Results

### CSF mtDNA in patients with NMOSD

The detection of extracellular mtDNA has been reported in various literatures by using qPCR method. Thus, mtDNA levels in CSF of NMOSD patients in acute phase were initially compared with those in patients with ONDs (GBS, ALS, iNPH, cervical spondylosis, and somatic symptom disorders) by analyzing the copy numbers in qPCR system (Tables 1 and 2). The level of CSF mtDNA was significantly higher in patients with NMOSD than in those with ONDs (*COX3*: *p* < 0.001; *ND1*: *p* < 0.001; *ND6*: *p* < 0.001) (Fig. 1a–c). In addition, the level of mtDNA was significantly higher in the relapse phase of NMOSD than in that of MS (*COX3*: *p* = 0.044; *ND1*: *p* = 0.042; *ND6*: *p* = 0.018) (Fig. 1a–c). Notably, the total amount of DNA was not significantly elevated in the acute CSF of NMOSD patients (Table 1, Fig. 1d). Furthermore, mtDNA levels among patients with similar CSF cell counts were also significantly higher in patients with NMOSD than the control group (see Additional files 1 and 2).

**Table 1:** Demographic features of patients and CSF findings

	NMOSD		MS	ONDs
	Relapse	Post-treatment	Relapse	
*n*	26	20	18	50
Age (years, range)	49.2 (26–74)	50.9 (26–74)	38.3 (18–64)	53.9 (15–83)
Female/Male	22/4	15/5	16/2	25/25
CSF cell counts (/mm^3^, range)	43.6 (0–423)	4.5 (0–14)	2.6 (0–11)	1.5 (0–16)
CSF protein (mg/dl, range)	51.1 (20–115)	33.6 (19–63)	36.7 (20–106)	41.6 (18–112)
Total amount of DNA (ng/100 μl CSF, range)	388 (142–1022)	351 (120–652)	442 (100–664)	392 (150–752)

*NMOSD* neuromyelitis optica spectrum disorder, *MS* multiple sclerosis, *ONDs* other neurological diseases, *CSF* cerebrospinal fluid

**Table 2:** Clinical characteristics of patients with NMOSD and MS in relapse phase

	NMOSD (*n* = 26)	MS (*n* = 18)
Disease duration, months (range)	42.7 (0–240)	62.4 (0–204)
Period from the acute relapse to CSF sampling, days (range)	14 (1–30)	19.2 (1–60)
Location of lesion		
Spinal cord (%)	23 (88.4)	5 (27.7)
Brain (%)	2 (7.6)	14 (77.7)
Optic nerve (%)	2 (7.6)	2 (11.1)
Length of spinal cord involvement, number of segments (range)	5.8 (2–17)	1.4 (1–2)
Treatment at CSF sampling (%)	7 (26.9)	11 (61.1)
Prednisolone	6 (23.0)	–
Immunosuppressants	4 (15.3)	–
Interferon-β	–	7 (38.8)
Fingolimod	–	3 (16.6)
Dimethyl fumarate	–	1 (5.5)

*NMOSD* neuromyelitis optica spectrum disorder, *MS* multiple sclerosis, *CSF* cerebrospinal fluid

Together, these results suggested that an elevated level of CSF mtDNA is a unique feature of NMOSD and not simply reflecting the increased CSF cell number.

### Treatment effect on CSF mtDNA level in NMOSD

**Fig. 2:**
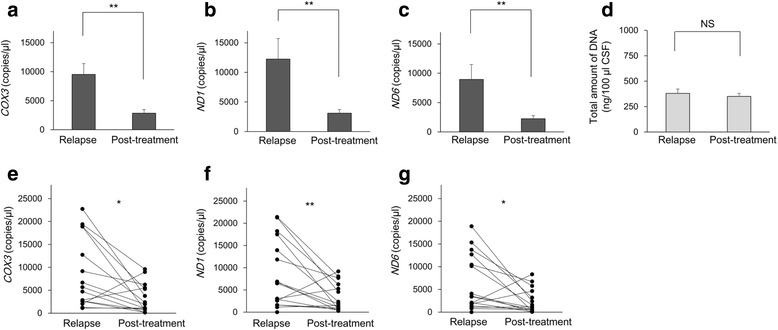
Effect of treatment on CSF mtDNA level in NMOSD. The mtDNA level was significantly higher in the relapse phase of NMOSD than in the post-treatment phase (**a**–**c**). The total amount of DNA did not differ significantly between the two groups (**d**). The mtDNA levels in paired samples in pre- and post-treatment phase were also significantly different (**e**–**g**). The data are expressed as means ± SEM. *p < 0.05; **p < 0.01; NS not significant (*p* ≥ 0.05)

To determine whether mtDNA levels are influenced by treatment of acute relapses, we examined mtDNA levels in NMOSD patients in relapse and post-treatment phases. The mtDNA level was significantly higher in the relapse phase of NMOSD than in the post-treatment phase (*COX3*: *p* = 0.004; *ND1*: *p* = 0.006; *ND6*: *p* = 0.006) (Fig. 2a–c). By contrast, the total amount of DNA did not significantly differ between the two groups (Fig. 2d). mtDNA levels of paired samples in pre- and post-treatment phase (*n* = 15) were also significantly different (*COX3*: *p* = 0.012; *ND1*: *p* = 0.009; *ND6*: *p* = 0.015) (Fig. 2e–g).

### AQP4-Ab mediates extracellular mtDNA release from astrocytes

**Fig. 3:**
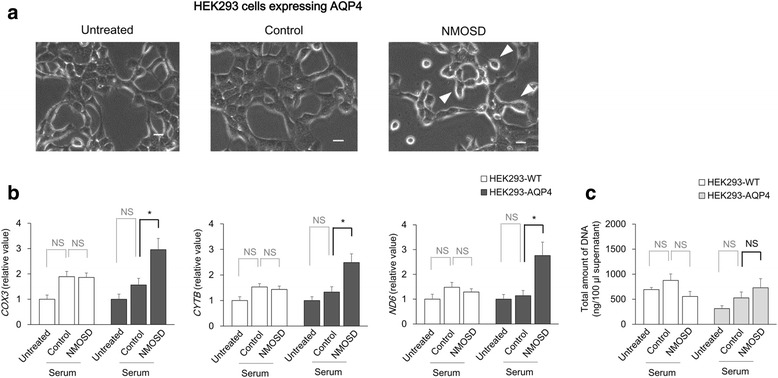
AQP4-Ab promotes extracellular mtDNA releases from HEK cells expressing AQP4. Cell cultures were stimulated with sera from NMOSD patients or healthy control subjects. HEK293 cells expressing AQP4 incubated with sera from NMOSD patients showed a balloon-like morphological change (arrowheads) (**a**). The level of extracellular mtDNA in the culture medium was higher with sera from NMOSD than with control sera in HEK293 cells expressing AQP4 (**b**). The total amount of DNA did not differ significantly between the two groups (**c**). The data are expressed as means ± SEM. *p < 0.05; NS not significant (*p* ≥ 0.05). Bar 20 m

**Fig. 4:**
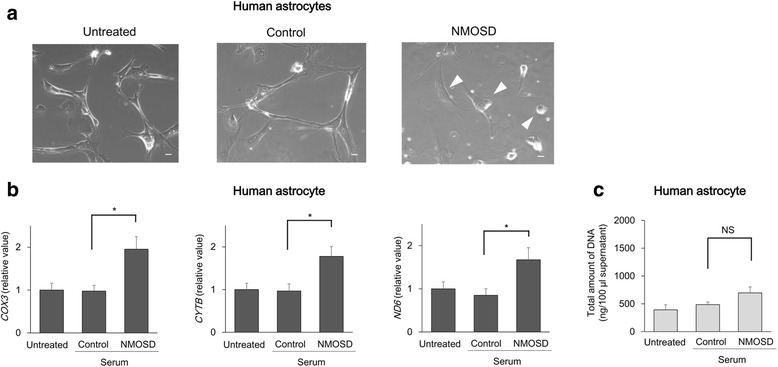
AQP4-Ab promotes extracellular mtDNA releases from astrocytes. Human astrocytes incubated with sera from NMOSD patients showed balloon-like morphological change (arrowheads) (**a**). The level of extracellular mtDNA in the culture medium was higher with sera from NMOSD in human astrocytes (**b**). The total amount of DNA did not differ between the two groups (**c**). The data are expressed as means ± SEM. *p < 0.05; NS not significant (*p* ≥ 0.05). Bar 20 μm

To investigate the specificity of AQP4-Ab-mediated mtDNA release, HEK293 cells expressing the *AQP4* gene were incubated with sera from NMOSD or healthy control subjects. Cells incubated with sera from NMOSD patients showed balloon-like morphological change (Fig. 3a), and the level of mtDNA in the cultured supernatant was significantly higher with sera from NMOSD patients than with sera from controls (*COX3*: *p* = 0.04; *CYTB*: *p* = 0.026; *ND6*: *p* = 0.026) (Fig. 3b). Moreover, sera from NMOSD patients did not affect mtDNA release from HEK293 cells not expressing human *AQP4* (Fig. 3b). Total DNA was not significantly altered by incubation with NMOSD sera (Fig. 3c). Human primary astrocytes incubated with NMOSD sera showed morphological change similar to HEK cells incubated with NMOSD sera (Fig. 4a) and exhibited specific mtDNA release in the supernatants (*COX3*: *p* = 0.016; *CYTB*: *p* = 0.036; *ND6*: *p* = 0.016) (Fig. 4b, c).

Taken together, these results indicated that AQP4-Ab specifically induces the extracellular release of mtDNA from astrocytes.

### Induction of IL-1β secretion from mixed glial cells by mtDNA

**Fig. 5:**
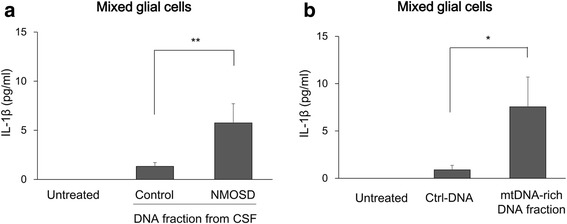
mtDNA promotes IL-1β secretion by mixed glial cells. Mixed glial cell cultures were stimulated with the DNA fraction containing abundant mtDNA. IL-1β secretion was promoted by the DNA fraction from CSF of NMOSD patients (**a**) or HEK293 cells expressing human AQP4 stimulated with sera from NMOSD patients (**b**). The data are expressed as means ± SEM. *p < 0.05; **p < 0.01

mtDNA activates innate immunity and induces the secretion of inflammatory cytokines [9]. Because the CSF of NMOSD patients contains abundant mtDNA, we stimulated mouse mixed glial cells with DNA fractions from CSF derived from NMOSD or OND patients for 48 h and examined the effect on cytokine production. DNA fractions isolated from CSF of NMOSD patients significantly promoted IL-1β secretion in comparison with the corresponding fractions from patients with ONDs (Fig. 5a). Notably, the amounts of total DNA isolated from each CSF sample did not significantly differ between the two groups (see Additional file 2). Similarly, DNA fractions isolated from culture media of AQP4-expressing HEK293 cells stimulated with NMOSD serum significantly promoted IL-1β production (Fig. 5b).

These data indicate that mtDNA released from astrocytes attacked by AQP4-Ab is capable of inducing IL-1β production by CNS glial cells.

### The TLR9 and NLRP3 pathways mediate IL-1β secretion

**Fig. 6:**
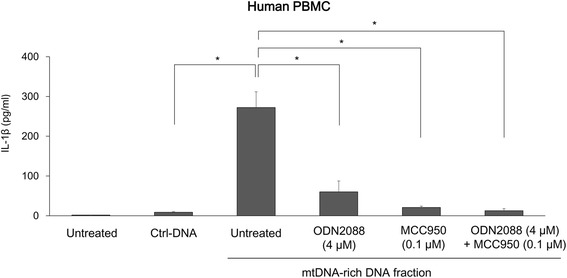
IL-1β production by DNA fraction containing abundant mtDNA is dependent on NLRP3 inflammasome and TLR9. IL-1β secretion was suppressed by inhibitors of the NLRP3 inflammasome (MCC950) and TLR9 (ODN2088) in PBMCs. The results are the representative of two independent experiments. The data are expressed as means ± SEM. *p < 0.05

To further elucidate the mechanism by which released mtDNA promotes the production of IL-1β in the pathogenesis of NMOSD, PBMCs stimulated with mtDNA isolated from culture media of AQP4-expressing HEK293 cells were co-incubated with specific inhibitors of TLR9 and NLRP3 inflammasomes. Both ODN2088, an inhibitor of TLR9, and MCC950, a specific inhibitor of NLRP3 inflammasomes, significantly inhibited IL-1β secretion from PBMCs stimulated with mtDNA. The effects of TLR9 and NLRP3 blockade were synergistic (Fig. 6).

## Discussion

In this study, we demonstrated that mtDNA is specifically elevated in the CSF at the acute phase of NMOSD. A previous study showed that mtDNA levels in CSF are elevated in relapsing-remitting MS (RRMS) patients in comparison to those with controls [15]. It was also reported that elevated mtDNA levels in CSF of MS patients are associated with higher T2 lesion volumes and lower normalized brain volume [16], suggesting that CSF mtDNA could serve as a marker reflecting the disease activity of MS. However, the association of mtDNA in NMOSD patients has not been previously demonstrated.

We found that the CSF mtDNA concentration was significantly higher in the acute phase of NMOSD than in MS patients. Cellular stress and necrosis are postulated to be major factors contributing to extracellular mtDNA release. In this regard, it is widely acknowledged that AQP4-Ab-mediated astrocyte necrosis leads to distinct lesion formation in NMOSD [17]. By contrast, MS pathology is characterized by demyelinating lesions infiltrated with inflammatory lymphocytes. Thus, the higher CSF mtDNA levels in NMOSD observed in our study presumably reflect the more destructive nature of NMOSD pathology, which is characterized by AQP4-Ab-mediated astrocyte damage.

Although the pathogenic mechanisms of both MS and NMOSD involve peripheral immune responses, NMOSD serum was capable of enhancing the release of mtDNA exclusively in HEK293 cells expressing AQP4, but not in control cells. The reproducibility of these results in studies utilizing human astrocytes further supported our hypothesis that AQP4-Ab-mediated astrocytic damage leads to pronounced release of mtDNA in the CSF of NMOSD patients.

In the context of therapeutic treatment, previous studies showed that RRMS patients treated with fingolimod have lower levels of CSF mtDNA in comparison with the baseline [16]. Consistent with this finding, we showed here that CSF mtDNA levels decline remarkably after the therapeutic period. These results imply that as in MS, CSF mtDNA levels could serve as a marker to reflect the disease activity of NMOSD. Although several studies have demonstrated the utility of glial fibrillary acidic protein (GFAP) as a marker for tissue damage in NMOSD pathology, it is important to note that CSF mtDNA not only reflects the extent of inflammation but also has the potential to induce innate immune responses by enhancing the section of IL-1β.

The role of mtDNA as a damage-associated molecular pattern (DAMP) that induces inflammation via innate immune receptors is well established [8, 9]. In this context, mtDNA has been shown to be involved in the pathogenesis of various diseases, including systemic inflammatory response syndrome (SIRS), HIV-associated dementia, and heart failure [8, 18, 19]. Consistent with these earlier findings, we found that the mtDNA-rich DNA fraction isolated from CSF of NMOSD patients increased IL-1β production from mixed glial cells.

Although we cannot exclude the possibility that nuclear DNA is involved in this inflammatory process, the amount of total DNA in the fraction isolated from CSF of NMOSD patients or culture media of an AQP4-overexpressing cell line incubated with AQP4-Ab did not differ significantly in comparison with the corresponding control. Thus, taken together, our data suggest that mtDNA is the major DNA fraction responsible for secretion of IL-1β from glial cells.

AQP4-Ab plays a central role in NMOSD by causing astrocyte necrosis. However, it remains unclear how astrocytic damage leads to secondary demyelination and neuronal loss. Recent studies reported that the levels of HMGB1, IL-1β, and IL-6 are elevated in the CSF of NMOSD patients [7, 20]. Interestingly in this regard, intracerebral IL-1β injection in the presence of AQP4-Ab triggers severe tissue damage in an animal model of NMOSD implying that innate immunity plays a pivotal role in the severe neural tissue damage associated with NMOSD [6]. Given that mtDNA released from astrocytes causes IL-1β secretion from macrophage/microglia, the activation of innate immunity by DAMPs including mtDNA represents a “missing link” between AQP4-Ab-induced astrocytic damage and severe inflammation leading to secondary demyelination and neural loss in NMOSD.

As for the innate receptors mediating IL-1β production, distinct activating pathways have been demonstrated between mouse and human [10, 11], further highlighting the importance of utilizing human-derived cells in the analysis of mtDNA-mediated pathogenesis. Here, we found extracellular release of mtDNA by NMOSD sera is consistently observed in human astrocytes and further showed the specific roles of innate receptors in human-derived PBMCs.

## Conclusion

We demonstrated that the level of CSF mtDNA is specifically elevated in the acute phase of NMOSD and further revealed that the extracellular mtDNA release by AQP4-Ab-mediated cellular damage elicits the innate immune cascades. Although the precise role of extracellular mtDNA in NMOSD pathogenesis observed in our study has to be clarified in vivo, our observation provides a novel therapeutic target for the treatment of NMOSD.

## Additional files

Additional file 1: **Figure S1.** mtDNA levels among patients of similar CSF cell counts. CSF cell counts and protein levels were not significantly different between NMOSD patients (*n* = 6) and controls (total *n* = 4: aseptic meningitis, *n* = 2; GBS, *n* = 1; neuropsychiatric systemic lupus erythematosus, *n* = 1) (cell counts (mean ± SEM): NMOSD, 33.8 ± 6.9/mm^3^; control, 25.0 ± 7.4/mm^3^) (protein (mean ± SEM): NMOSD, 53.3 ± 6.0 mg/dl; control, 72.2 ± 17.8 mg/dl) (**a**). mtDNA levels in CSF were significantly higher in patients with NMOSD than in controls (**b–d**). Total DNA amount was not significantly different between the two groups (**e**). *P < 0.05; NS not significant (*P* ≥ 0.05). (PDF 392 kb)

Additional file 2: **Table S1.** Clinical and CSF characteristics of patients with similar CSF counts. **Table S2.** Clinical and CSF characteristics of patients enrolled in the experiments utilizing DNA fractions from CSF. (PDF 56 kb)

## Abbreviations

ALS: Amyotrophic lateral sclerosis
ANCOVA: Analysis of covariance
AQP4-Ab: Anti-aquaporin 4 autoantibody
CNS: Central nervous system
COX3: Cytochrome C oxidase 3
CSF: Cerebrospinal fluid
CYTB: Cytochrome B
DMEM: Dulbecco’s Modified Eagle’s Medium
FBS: Fetal bovine serum
GBS: Guillain-Barré syndrome
IL: Interleukin
iNPH: Idiopathic normal pressure hydrocephalus
MS: Multiple sclerosis
mtDNA: Mitochondrial DNA
ND1: NADH dehydrogenase 1
ND6: NADH dehydrogenase 6
NLRP3: NOD-like receptor protein 3
NMOSD: Neuromyelitis optica spectrum disorder
OND: Other neurological disease
PBMC: Peripheral blood mononuclear cell
qPCR: Quantitative polymerase chain reaction
RRMS: Relapsing-remitting multiple sclerosis
TLR9: Toll-like receptor 9
