# Supplementary material for: Cerebrospinal fluid mitochondrial DNA in neuromyelitis optica spectrum disorder
Source: J Neuroinflammation. 2018 Apr 27;15:125. doi: 10.1186/s12974-018-1162-0 (PMC5924507; doi:10.1186/s12974-018-1162-0)
Supplement: Supplementary file 1 — Figure S1. mtDNA levels among patients of similar CSF cell counts. CSF cell counts and protein levels were not significantly different between NMOSD patients (n = 6) and controls (total n = 4: aseptic meningitis, n = 2; GBS, n = 1; neuropsychiatric systemic lupus erythematosus, n = 1) (cell counts (mean ± SEM): NMOSD, 33.8 ± 6.9/mm3; control, 25.0 ± 7.4/mm3) (protein (mean ± SEM): NMOSD, 53.3 ± 6.0 mg/dl; control, 72.2 ± 17.8 mg/dl) (a). mtDNA levels in CSF were significantly higher in patients with NMOSD than in controls (b–d). Total DNA amount was not significantly different between the two groups (e). *P < 0.05; NS not significant (P ≥ 0.05). (PDF 392 kb) [file 12974_2018_1162_MOESM1_ESM.pdf]

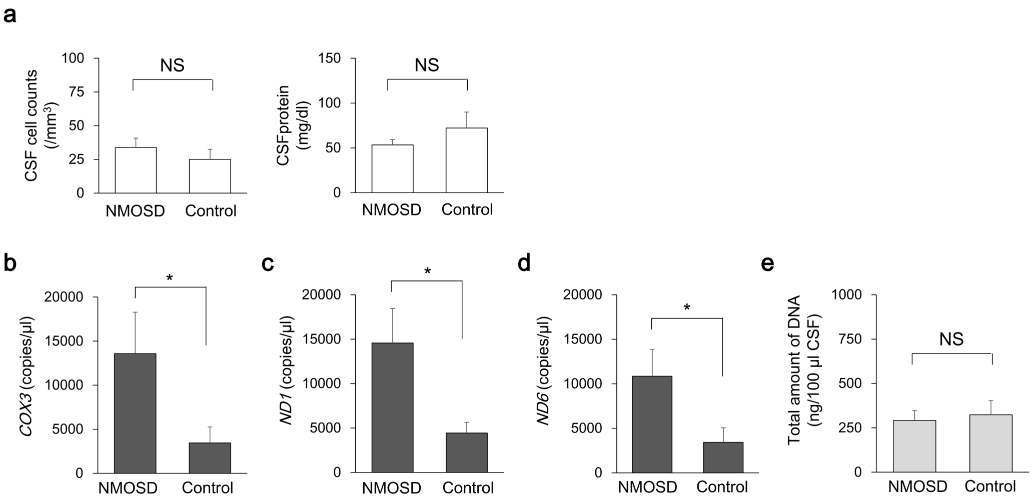

Fig. S1 mtDNA levels among patients of similar CSF cell counts.

CSF cell counts and protein levels were not significantly different between NMOSD patients (n=6) and controls (total n=4: aseptic meningitis, n=2; GBS, n=1; neuropsychiatric systemic lupus erythematosus, n=1) (cell counts (mean±SEM): NMOSD, 33.8±6.9 /mm^3; Control, 25.0±7.4 /mm^3) (protein (mean±SEM): NMOSD, 53.3±6.0 mg/dl; Control, 72.2±17.8 mg/dl) (**a**). mtDNA levels in CSF were significantly higher in patients with NMOSD than in controls (**b–d**). Total amount of DNA was not significantly different between the two groups (**e**). *$P<0.05$; NS, not significant ($P\geq0.05$).
