# Supplementary material for: Cerebrospinal fluid mitochondrial DNA in neuromyelitis optica spectrum disorder
Source: J Neuroinflammation. 2018 Apr 27;15:125. doi: 10.1186/s12974-018-1162-0 (PMC5924507; doi:10.1186/s12974-018-1162-0)
Supplement: Supplementary file 2 — Table S1. Clinical and CSF characteristics of patients with similar CSF counts. Table S2. Clinical and CSF characteristics of patients enrolled in the experiments utilizing DNA fractions from CSF. (PDF 56 kb) [file 12974_2018_1162_MOESM2_ESM.pdf]

Table S1. Clinical and CSF characteristics of patients with similar CSF counts.

Patients	Disease	Age (years)	Gender	CSF		Total amount of DNA (ng/100μl CSF)
				Cell counts (/mm^3)	Protein (mg/dl)	
1	NMOSD	44	F	55	62	252
2	NMOSD	55	F	18	65	246
3	NMOSD	38	M	41	49	444
4	NMOSD	73	M	46	42	476
5	NMOSD	50	F	33	70	142
6	NMOSD	43	F	10	32	194
7	aseptic meningitis	76	F	47	113	244
8	aseptic meningitis	57	F	22	68	146
9	GBS	40	F	16	81	450
10	NPSLE	33	F	15	27	474

NMOSD = neuromyelitis optica spectrum disorder; GBS = Guillain-Barré syndrome; NPSLE = neuropsychiatric systemic lupus erythematosus; F = female; M = male.

Table S2. Clinical and CSF characteristics of patients enrolled in the experiments utilizing DNA fractions from CSF.

Patients	Disease	Age (years)	Gender	CSF		Final concentration	
				Cell counts (/mm^3)	Protein (mg/dl)	Total DNA (ng/μl)	mtDNA (copies/μl)
1	NMOSD	39	F	64	45	4.9	1244.0
2	NMOSD	55	F	18	65	4.2	2076.1
3	NMOSD	26	F	80	73	1.1	4717.5
4	NMOSD	63	F	3	27	5.6	1075.3
5	NMOSD	73	F	46	42	2.4	1426.2
6	iNPH	79	F	1	38	3.0	121.6
7	iNPH	64	F	0	40	2.0	57.3
8	iNPH	75	F	0	65	3.3	83.9
9	Anti-NMDA receptor encephalitis	31	F	9	34	6.9	42.3
10	Somatic symptom disorder	36	M	1	28	7.8	41.8
11	ALS	67	F	0	20	3.6	85.8

NMOSD = neuromyelitis optica spectrum disorder; iNPH = idiopathic normal pressure hydrocephalus; ALS = amyotrophic lateral sclerosis; F = female; M = male.
